# School of Anatomy and Medicine Adjoining St. George’s Hospital

**Published:** 1852-11

**Authors:** 


School of Anatomy and Medicine, adjoining St. George's Hospital. *Winter Session.*—Anatomy and Physiology, Messrs. Lane, Blenkins, and J. R. Lane. Descriptive and Surgical Anatomy, Messrs. Blenkins, and J. R. Lane. Chemistry, Mr. Rodgers. Medicine, Drs. Daniell and Sibson. Surgery, Messrs. Pilcher and Smith. *Summer Session.*—Materia Medica and Therapeutics, Dr. Lankester. Midwifery, Mr. Bloxam. Medical Jurisprudence, Mr. Warder. Botany, Dr. Lankester. Practical Chemistry, Mr. Rodgers.

